# Importance of National Fitness Sports Relying on Virtual Reality Technology in the Development of Sports Economy

**DOI:** 10.1155/2022/4128981

**Published:** 2022-08-25

**Authors:** Lianzhen Chen, Hua Zhu

**Affiliations:** ^1^School of Sports Science, Lingnan Normal University, Zhanjiang, Guangdong 524048, China; ^2^Physical Education Department, Guangdong Medical University, Zhanjiang, Guangdong 524023, China

## Abstract

With the rapid development of the national economy and the improvement of people's living standards, physical fitness and health have attracted people's attention and become an important topic in people's daily life. Virtual reality technology is a new technology that uses computer to build virtual environment, belonging to simulation technology, which is an extremely important direction in this field. This paper aims to study the importance of the national fitness sports relying on virtual reality technology in the development of sports economy. The related concepts of virtual reality technology and the related meanings of national fitness are introduced. The characteristics of the office population are analyzed to understand the daily behavior habits, health status, and common diseases of the target population. Meanwhile, the traditional home fitness equipment and virtual home fitness equipment are collected and compared. The problems of the existing home fitness equipment are understood so as to discover the direction of subsequent design. Then, through interviews, questionnaires, and on-the-spot research, the target users and the product usage environment are investigated in depth. The pain points and needs of users are found and the needs of office workers for fitness products are summarized. The experimental results showed that in the national fitness sports relying on virtual reality technology, 80% of the respondents believed that the integration of somatosensory technology into fitness equipment has a positive effect on improving fitness.

## 1. Introduction

With the rapid development of science and technology, the continuous development of virtual reality technology has greatly affected people's lives. People are no longer the same as before. They were outside the scene for the various scenes and pictures created by electronic computers. This new technology also plays a huge role in helping people understand their self-awareness. Virtual reality technology uses computer software to create a virtual simulation environment, which is interactive; that is, users can see a three-dimensional vision in the virtual environment that is very similar to the real world and can use wearable devices to generate physical behaviors. Virtual reality technology involves many existing technologies, such as sensor technology and multimedia technology, which has a very close connection with graphics and other disciplines. The research and development of this technology is extremely challenging.

Physical fitness refers to the sports activities that aim to enhance people's physical fitness, improve people's health level, and cultivate people's good sentiments by exercising with only hands or using various equipment and specialized and scientific movement methods. For people who are currently in subhealth state who sit in front of the computer for a long time and work, physical fitness has more important practical significance. The innovation of this paper lies in the philosophical research on virtual reality technology, which helps to guide this medium technology to better serve human society. Any technology is a double-edged sword. Avoiding its negative effects in the process of application and giving full play to its benign effects are the problems that need to be solved now. By analyzing the epistemology and practice theory of virtual reality technology, and exploring its influence on society, it helps people to realize the importance of virtual reality technology so that human beings and media technology can grow in harmony, and the virtual world and the real world can complement each other.

## 2. Related Work

As people gradually attach importance to fitness, more and more scholars have begun to study national fitness. The purpose of the Kidokoro et al.'s study [1] was to assess temporal trends in balance and walking speed in elderly Japanese people (65–79 years old) between 1998 and 2018 [1]. Wang et al. [2] studied the association of estimated cardiorespiratory function (eCRF) with all-cause and cardiovascular disease (CVD) mortality in a representative population of the United States [2]. The study of Talbot et al. [3] investigated the effects of a brain matter dementia risk reduction exercise in Australia [3]. The purpose of Jhang et al. [4] research was to promote community health, with a focus on older community residents. The effects of lower extremity exercise intervention on functional health, physiological indicators, exercise self-efficacy, sleep quality, and mental health of middle-aged and elderly people were discussed [4]. However, the shortcoming of these studies is that the models constructed are not scientific enough.

In recent years, research activities in the field of virtual reality have been very active. Aliyu and Talib [5] discussed the recent application of virtual reality technology (VRT) in educational settings and self-learning of chemistry, especially considering that preservice chemistry teachers in Nigeria need an effective strategy for learning chemistry concepts [5]. Maples-Keller et al. [6]reviewed the history of VR-based technology and its application in psychiatric care, empirical evidence for VR-based treatment, and the benefits of using VR for psychiatric research and treatment [6]. Liang and Shuang [7] conducted research on value identification and traditional village protection based on virtual reality technology [7]. Zhang and Zheng [8] further studied two key issues in real-time rendering to achieve and improve the modeling speed of virtual walkthroughs [8]. The shortcomings of these studies are that some theoretical studies are not perfect, and there are still many practical problems to be solved.

## 3. Relevant Methods of National Fitness Sports Relying on Virtual Reality Technology

### 3.1. Virtual Reality Technology

#### 3.1.1. Concept of Virtual Reality Technology

Virtual reality (VR for short) is a technology first proposed by the United States in the early 1980s. Virtual means virtual, and reality means real; thus, the combination is virtual reality. Virtual reality technology is based on 3D production technology, graphics rendering technology, and sensor technology, and it finally generates a virtual scene after processing. Through the fusion of this virtualized and three-dimensional multisource information, people can feel the virtual world just like the real world, which is the virtual reality technology [9, 10]. Virtual reality technology is recognized as one of the important development disciplines in the twenty-first century and one of the important technologies affecting human life.

In the virtual environment, the user interacts with the objects in the virtual environment through VR glasses and handles, and affects the objects in the virtual environment, so that the user feels that they are in another world.

#### 3.1.2. Types of Virtual Reality Systems

According to the level of immersion and the degree of interaction, VR systems can be divided into the following four types:

*(1) Desktop VR System*. A desktop virtual reality system (desktop virtual reality), referred to as DVR for short, presents a three-dimensional space on a computer monitor or other display screen and uses it as a window for a user to observe a virtual scene. In the real world, the experiencer can use the mouse, data gloves, and other devices to interact with the virtual scene on the screen [11].

In the DVR system, because the user is in a real environment, it is easier to be disturbed by the outside world, and it is difficult to fully immerse in the virtual world. However, the DVR system has low cost and simple structure, which is easily accepted by the public. It is an economical and practical virtual reality system.

*(2) Augmented VR System*. Augmented VR system, referred to as augmented reality (AR), uses computer technology to superimpose objects in the virtual world on the real world through the screen. In this way, users can see computer-generated virtual objects based on the real environment while seeing the real-world environment [12]. Augmented VR systems have the following three characteristics: “seamless” integration of real and virtual world information, real-time interactivity, virtual world and real world integration in three-dimensional space.

*(3) Immersive VR System (Immersive VR)*. Immersive VR systems use various interactive devices such as head-mounted displays and data gloves to temporarily separate users from the real world, so that the users are completely immersed in the virtual world without interference from the real world, achieving an immersive effect. The monitor will display the impact of the experiencer's actions on the virtual environment in real time so as to achieve the effect of real-time interaction.

*(4) Distributed Virtual Reality System (Distributed VR)*. The distributed virtual reality system uses computer networks to connect users in different regions to the same virtual environment through the Internet. In this way, although everyone is everywhere, they can experience the same virtual world and achieve the same daily work or entertainment.

#### 3.1.3. Main Features of Virtual Reality Technology

*(1) Interactivity*. Virtual reality technology is interactive [13]. The interaction between the user and the virtual environment is through VR glasses and VR controllers. The user manipulates the objects in the virtual environment through the handle, and the VR glasses finally display the result of the user manipulation through a series of algorithms. This allows users to experience the interaction between themselves and the virtual environment.

*(2) Immersion*. In the virtual reality system, the virtual scene generated by computer simulation will be displayed on the screen or other output devices. At the same time, it will be matched with the corresponding sound, picture, etc. On the other hand, the experiencer will have a real tactile sensation by manipulating the input device. The virtual reality system integrates visual, auditory, tactile, and other human perceptions to bring the experiencer a sense of immersion close to the real world.

*(3) Imagination*. Imagination means that when the experiencer is immersed in the virtual scene, he will think and infer the picture presented by the virtual scene and then generate associations, combining the virtual scene with the imagination to achieve a more realistic experience, which can further stimulate new associations. Because in the virtual reality system scenes and things that cannot be seen in the real world can be presented, the virtual reality technology is conducive to cultivating the imagination and creativity of the experiencer.

#### 3.1.4. Technical Composition and Principle

**Figure 1:**
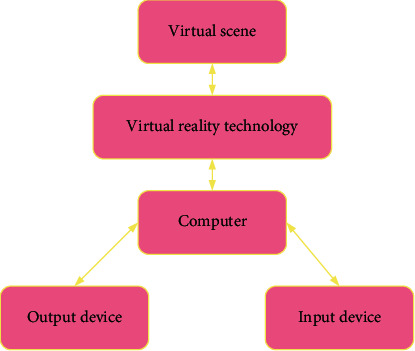
Composition of the virtual reality system.

A virtual reality system is a computer simulation technology, generally composed of computer hardware equipment, virtual reality technology carrying software, input equipment, and output equipment, as shown in Figure 1 [14]. VR provides a method to develop realistic virtual environments with the advantage of allowing better control of experimental conditions while conferring good ecological validity.

**Figure 2:**
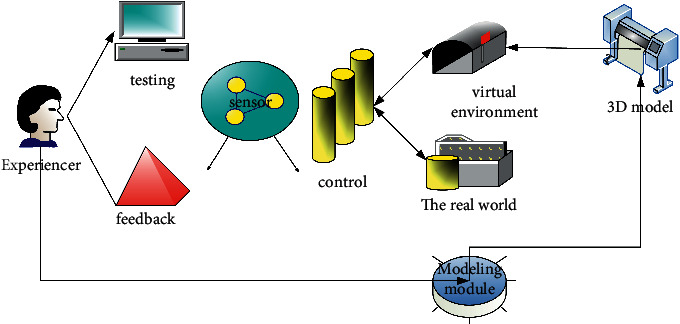
Principle of virtual reality technology implementation.

Virtual reality technology refers to the technology in which the experiencer produces interactive behaviors in the real space through the virtual scene generated by the combination of sensor collection equipment, computer hardware, and a VR engine. The realization principle is that in the 3D model created by the computer, the original data of the user are captured by the sensor, and the computer hardware processes and extracts the original data to obtain the behavior data. Then it is input into the virtual environment, and finally the application changes generated in the virtual environment are fed back to the sensor to realize the interaction process between the virtual scene and the experiencer, as shown in Figure 2 [15].

#### 3.1.5. Classification

**Figure 3:**
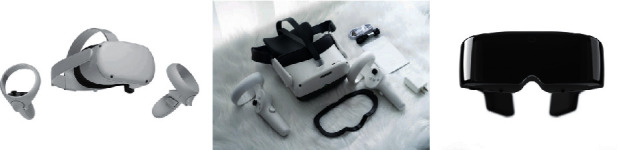
Virtual reality hardware device. (a). Head-mounted VR. (b). All-in-one VR. (c). VR glasses.

Due to different VR hardware devices, the VR presentation mode is usually divided into fixed-side VR (interactive experience mode) and mobile-side VR (360° panoramic photo mode). Fixed-end VR refers to external computers and professional sensor devices represented by Oculus Rift, Samsung Xuanlong, and HTC. Mobile VR refers to the use of VR glasses and mobile phones as players, as shown in Figure 3. In Table 1, the differences, advantages, and disadvantages of the devices are compared.

In fixed-side VR, the user can directly interact with the design program because the device can capture the activity state of the experiencer and feed it back into the VR environment [16]. This enables users to evaluate designs more accurately and have a more in-depth experiential experience. Mobile VR uses cloud platforms and mobile phone software to view 360° panoramic images. The synthesis of panoramic images mainly comes from three ways: ordinary camera shooting, software postsynthesis, and panoramic camera shooting. The advantages are that the experience is portable and the price is relatively cheap. But the disadvantage is that it largely depends on the performance of the mobile phone. The higher the resolution of the mobile phone is, the better the picture effect and experience will be. There is a certain gap compared with the real immersive experience of fixed-end VR. To some extent, it can be argued that mobile VR is not VR in the full sense.

### 3.2. National Fitness Exercise Based on Virtual Reality Technology

#### 3.2.1. Concept of National Fitness

The definition of national fitness under the Internet query is as follows: National fitness refers to the people of the whole country regardless of gender or age, can make all the people strengthen their power, increase their flexibility and endurance, and improve their coordination, so as to make the people physically strong [17]. It is believed that national fitness is a systematic project, calling on the people of the whole country to carry out physical exercise and improve the overall quality of the people.

#### 3.2.2. Analysis of the Kinect-Based Human Motion Capture Algorithm

The Kinect camera can not only collect color and depth images but also detect and track 25 joints of the human body in real time at a frequency of 30 frames per second and return their 3D spatial coordinates to obtain the unmarked spatial position of human joints. The depth information captured by the Kinect camera has a wide range of applications in gesture recognition, action recognition, and 3D reconstruction [18]. This section mainly describes the principle of depth image and bone tracking technology, with the details of the method of hand joint position inference.

*(1) Extraction of Arm Depth Images*. Usually the depth value of the torso part of the human body is the largest in the depth information statistics, so the deleted part can be defined as (assuming there are *s* depth blocks)

(1) $$\begin{array}{c} p\in \zeta ,\text{Depth}\left(p\right)=\underset{1\leq q\leq S}{\max\left\{\text{Depth}\left(K_{\text{d}}\right)\right\}}. \end{array}$$

In formula (1), *ζ* is the part other than the arm; *p* is a pixel of the depth block in the depth image.

The body part (region *X*) and the arm part (region *Y*) are divided by choosing a threshold. For this histogram, it is supposed there are *G* depth values, and the sum of the remaining depth blocks is *N*. The number of depth blocks with depth value *i* is *n*, and each depth value occurs with probability *Q*. Formulas (2) and (3) can be obtained as

(2) $$\begin{array}{c} N=\sum_{i=0}^{G-1}n, \end{array}$$

(3) $$\begin{array}{c} Q_{i}=\frac{n_{i}}{N}, \end{array}$$

The occurrence probability of region *X* and region *Y* satisfies formulas (4) and (5):

(4) $$\begin{array}{c} Q_{X}=\sum_{i=t+1}^{G-1}Q_{i}, \end{array}$$

(5) $$\begin{array}{c} Q_{Y}=\sum_{i=0}^{t}Q_{i}, \end{array}$$

Among them, *t* is the assumed selected threshold. The between-group variances of region *X* and region *Y* satisfy formulas (6)–(8):

(6) $$\begin{array}{c} \omega_{X}=\sum_{i=t+1}\frac{iQ_{i}}{Q_{X}}, \end{array}$$

(7) $$\begin{array}{c} \omega_{Y}=\sum_{i=0}^{t}\frac{iQ_{i}}{Q_{Y}}, \end{array}$$

(8) $$\begin{array}{c} \delta^{2}\left(t\right)=Q_{X}{\left(\omega_{X}-\omega_{O}\right)}^{2}+Q_{Y}{\left(\omega_{Y}-\omega_{O}\right)}^{2}. \end{array}$$

In formulas (6)–(8), *ω*_*X*_ is the average depth value of region *X*; *ω*_*Y*_ is the average depth value of region *Y*; and *δ*^2^(*t*) is the variance of the two regions, representing a measure of the uniformity of the depth distribution.

The extraction of the arm part is based on the feature of depth information to select the threshold value so as to maximize the variance between region *X* and region *Y*. If the variance between the two regions is too small, the extracted image will contain too much body depth information, resulting in the extraction failure. Therefore, the largest variance means the smallest probability of misclassification. The optimal segmentation threshold should maximize the variance of region *X* and region *Y*. The optimal threshold *R* should satisfy formula (9):

(9) $$\begin{array}{c} R=\underset{0\leq i\leq G-1}{\max}\left\{\delta^{2}\left(t\right)\right\}. \end{array}$$

**Figure 4:**
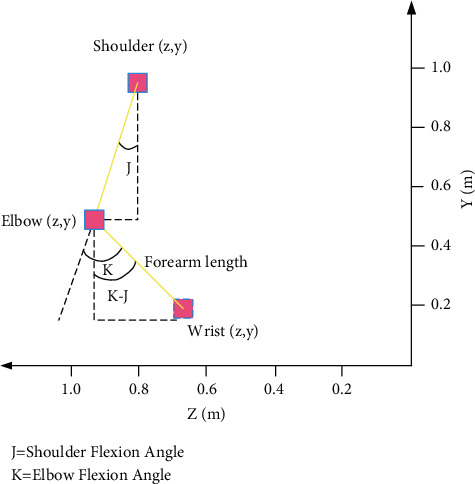
Schematic diagram of the relationship between the human arm joints in the *YZ* plane.

*(2) Inference of Hand Joint Position*. To infer the correct hand joint positions from depth images, it is first necessary to improve the correct recognition rate of the wrist joints [19]. This paper proposes a method for inferring the wrist joints; that is, in the process of swinging the arms, the position of the wrist joint is determined by the flexion and extension angles of the shoulders and elbows. The angle and space relationship between the joints are shown in Figure 4. The calculation formulas of the wrist space coordinates are given as

(10) $$\begin{array}{c} \text{Wrist}\left(z\right)=\text{Elbow}\left(z\right)+\text{sin}\left(K-J\right)\text{Length,} \end{array}$$

(11) $$\begin{array}{c} \text{Wrist}\left(y\right)=\text{Elbow}\left(y\right)-\text{cos}\left(K-J\right)\text{Length,} \end{array}$$

(12) $$\begin{array}{c} \text{Wrist}\left(x\right)=\text{Elbow}\left(x\right). \end{array}$$

Wrist (*x*), Wrist (*y*), and Wrist (*z*) represent the *x*, *y*, and *z* values of the wrist. Elbow (*x*), Elbow (*y*), and Elbow (*z*) represent the *x*, *y*, and *z* values of the elbow. Length is the length of the forearm, that is, forearm length. Angle *J* is the angle at which the upper arm is bent and extended. Angle *K* is the angle of flexion and extension of the forearm.

After determining the positions of the elbow and wrist joints, the depth image of the arm is segmented twice, and only the depth image of the hand is retained. The image is converted into a binary image, and the coordinates of the hand joints are obtained using the centroid formula. It is supposed that the binary image is *F* (*x*, *y*), and the target part is *J*. The centroid (*X*_0_, *Y*_0_) of the target is defined as

(13) $$\begin{array}{c} F\left(x,y\right)=\left\{\begin{array}{cc} 1, & \left(x,y\right)\in J,\\ 0, & \left(x,y\right)\in J, \end{array}\right) \end{array}$$

(14) $$\begin{array}{c} x_{0}=\frac{\sum_{\left(x,y\right)\in J}xF\left(x,y\right)}{\sum_{\left(x,y\right)\in J}F\left(x,y\right)}, \end{array}$$

(15) $$\begin{array}{c} y_{0}=\frac{\sum_{\left(x,y\right)\in J}yF\left(x,y\right)}{\sum_{\left(x,y\right)\in J}F\left(x,y\right)}. \end{array}$$

### 3.3. Experiment and Deconstruction of National Fitness Sports Relying on Virtual Reality Technology

#### 3.3.1. Investigation and Deconstruction of Product Design for Virtual Entertainment and Fitness

This chapter mainly obtains users' needs for virtual reality fitness equipment through interviews and user questionnaires so as to provide accurate design directions for the next design [20, 21].

Purpose of the interview: The target users' work status, physical condition, fitness status, and other issues were understood and problems were found from it. Thus, the design entry point was found, and the interview results provided help for subsequent larger-scale questionnaires.

User sample selection: This project took sedentary office workers as the target group. When selecting interviewees, comprehensive consideration was given to age, occupation, and living conditions. A total of six target users were found for interviews.

Interview process and method: The interview outline was prepared in advance according to the research content, and the key questions were marked. Before the start of the interview, the purpose of the interview was explained and self-introduction was given, and then questions were asked according to the outline. The questions were adjusted according to the respondents' answers to achieve the purpose of the interview [22]. The content of the interview was recorded by a combination of notes and audio recordings, which facilitated the statistics of the results after the interview.

#### 3.3.2. Arrangement and Analysis of Interview Content

*(1) Interviews on the Basic Situation of Users*. It can be seen from Table 2 that the six respondents had shoulder and neck problems after working at a desk for a long time. However, they did not carry out professional treatment or adjustment after the problem occurred, nor did they know how to adjust, with a lack of professional guidance.

It can be seen from Table 3 that the six respondents exercised regularly, but the frequency of weekly fitness was low. Because of their workdays, their exercise time was basically concentrated in the evening or on weekends. Most of the fitness methods were simple projects such as running or doing sit-ups and push-ups at home. Two of the respondents would search for relevant fitness videos on the Internet for follow-up exercises. In the choice of fitness venues, the six respondents exercised in the downstairs of the community, nearby parks, and homes, and only one person chose to go to the gym for exercise.

*(2) Interview about Fitness Style*. When conducting interviews on fitness methods, the respondents all expressed their wish to go to the gym for professional fitness training. For example, male respondents hoped to go to the gym to use professional equipment for exercise, and female respondents hoped to go to the gym to obtain professional fitness and movement guidance (as shown in Table 4), but the six respondents rarely went to the gym in real life. On the one hand, it was because there was no time to go to the gym, and the journey was long. On the other hand, the cost of going to the gym for exercise was high and could not be afforded.

*(3) Opinion Interview about Adding Virtual Somatosensory Games to Fitness*. Most users found it more interesting when virtual games were added to fitness. However, there were different opinions on whether somatosensory games were effective in relieving shoulder and neck cone disease (as shown in Table 5).

#### 3.3.3. Summary of Interview Results

Based on the collation and analysis of the interview results, the author summarizes the needs of the interviewees as follows:

*(1) Convenience Requirements*. Since the target groups were office workers and part-time workers working in the office, working time occupied a large part of daily life, and there was not enough time to go to the gym or exercise outdoors. Therefore, they preferred to use some fragmented time to exercise at home and hoped to have a fitness equipment that could be used for physical exercise at home at any time, which was convenient, time-saving, and efficient to exercise.

*(2) Professional Requirements*. The target users of this research were young working groups of age 20–35 years. They had a high level of education and had certain requirements for scientific and professional fitness methods. However, because they lacked the time to collect and learn relevant professional fitness knowledge, when designing, the guidance function of the product should be increased to adapt to the fitness needs of different target groups so as to provide users with scientific fitness guidance and functional correction services.

*(3) Social Requirements*. Humans are social animals. As an office worker with long-term work and greater psychological pressure, it is more necessary to socialize to relieve mental pressure and improve mood. Therefore, they hoped that home fitness products could have social functions, which was convenient for them to communicate and interact with friends or others on fitness and to stimulate their interest in fitness so that they could keep exercising.

*(4) Entertainment Requirements*. Traditional fitness methods are generally boring and monotonous. Especially when the target users were exercising at home, they felt it more boring because they are exercising alone. Therefore, they had entertainment needs for fitness products and hoped that they could also have entertainment during fitness, enriching the fitness experience and improving the emotional experience so that they could exercise happily.

### 3.4. Questionnaire Investigation

#### 3.4.1. Purpose of the Questionnaire Investigation

The study investigated more about the target user's health, fitness style, fitness frequency, and fitness needs. The number and scope of the research objects have been expanded, and more general data have been mastered to provide ideas and help for the subsequent design of home fitness products.

#### 3.4.2. Designing and Formulating Questionnaires

The questionnaire was divided into three parts. The first part was the collection of basic user information. The second part was the survey of the respondents' physical condition and fitness status. The third part was a survey of users' opinions on somatosensory games.

#### 3.4.3. Collation and Analysis of Questionnaire Results

A total of 150 questionnaires were distributed in this survey, and 138 valid questionnaires were returned. The effective rate of the questionnaires was about 92%. Among them, women accounted for 58% and men accounted for 42%, which met the statistical requirements. The following is the statistics and analysis of the questionnaire results.

**Figure 5:**
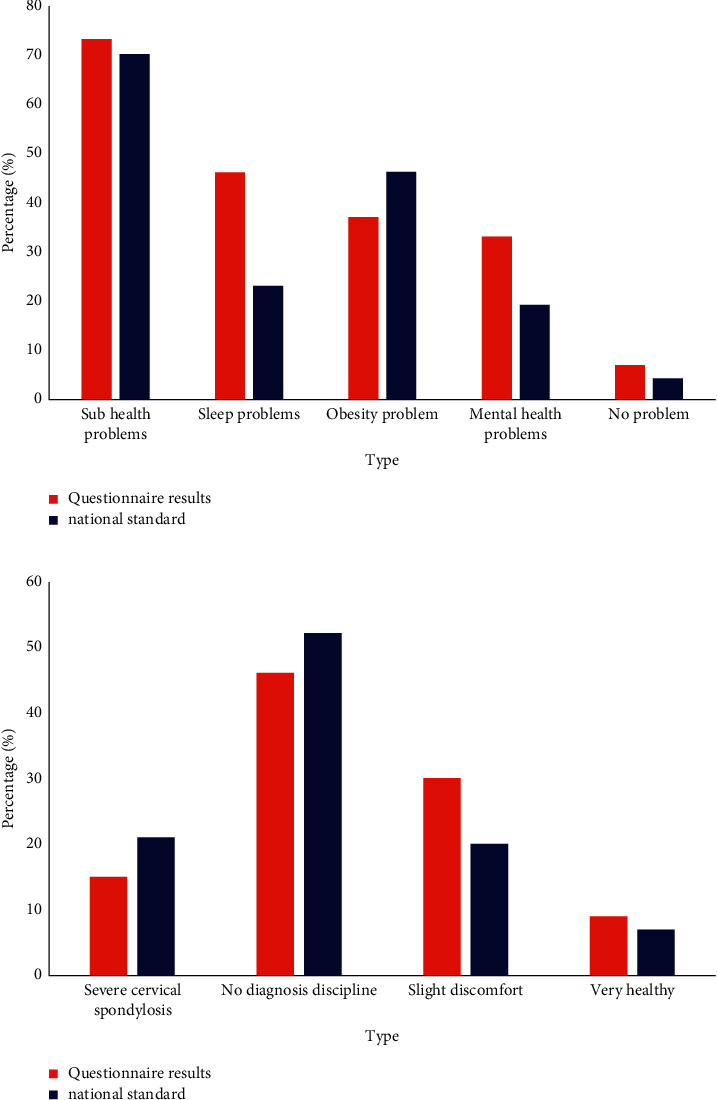
Health status. (a). Physical problems. (b). Basic conditions of the shoulder and neck.

*(1) Health Status*. It can be seen from the questionnaire results that in terms of health status, 73% of the respondents had subhealth conditions such as cervical spine pain and lumbar spine discomfort. In the sleep quality survey, 46% of the respondents had sleep problems, such as insomnia. Only 7% of all respondents believed that they did not have health problems, which showed that the health problems of office workers were needed to be paid attention to. The results of the survey on the specific health status of the respondents are shown in Figure 5(a).

*(2) Basic Condition of Shoulder and Neck*. It can be seen from Figure 5(b) that most of the subjects had discomfort in the shoulder and neck. Most people did not pay too much attention to these problems. Some of the more serious cases would go to the hospital for inquiry and treatment.

**Figure 6:**
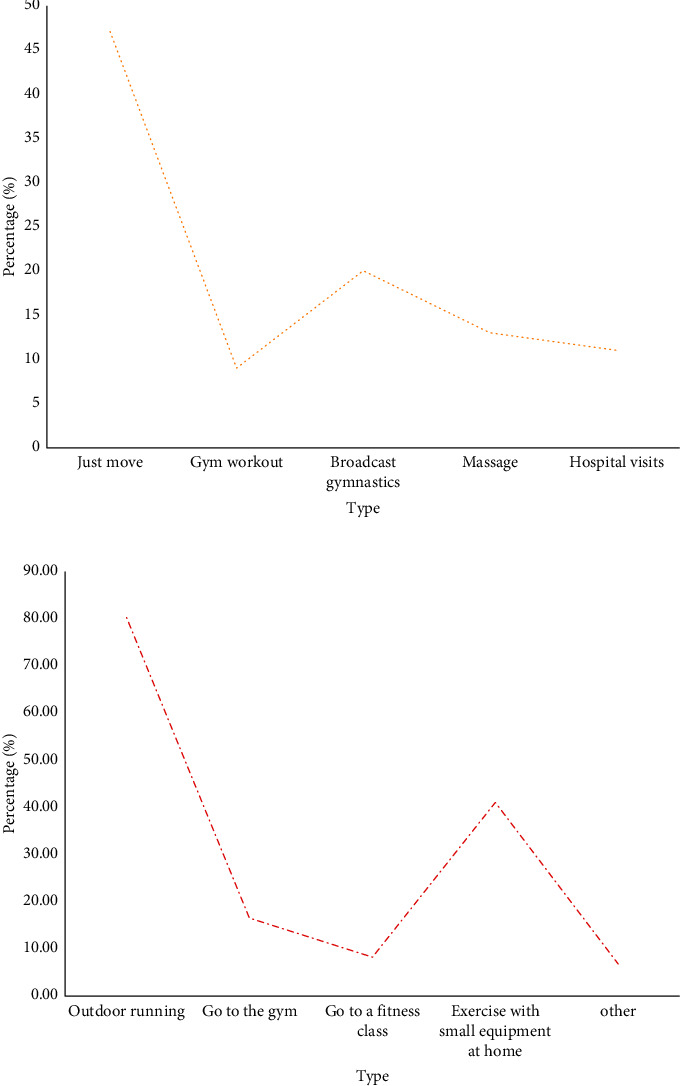
Measures and fitness methods after sitting for a long time and feeling uncomfortable. (a) Measures to be taken after prolonged sitting and discomfort. (b). Fitness methods.

From the perspective of fitness methods (as shown in Figure 6(a)), most people exercised outdoors or used small equipment at home to exercise. Few people went to professional gyms or yoga studios to exercise.

From the perspective of fitness methods (as shown in Figure 6(b)), most people exercised outdoors or used small equipment at home to exercise. Few people went to professional gyms or yoga studios to exercise.

**Figure 7:**
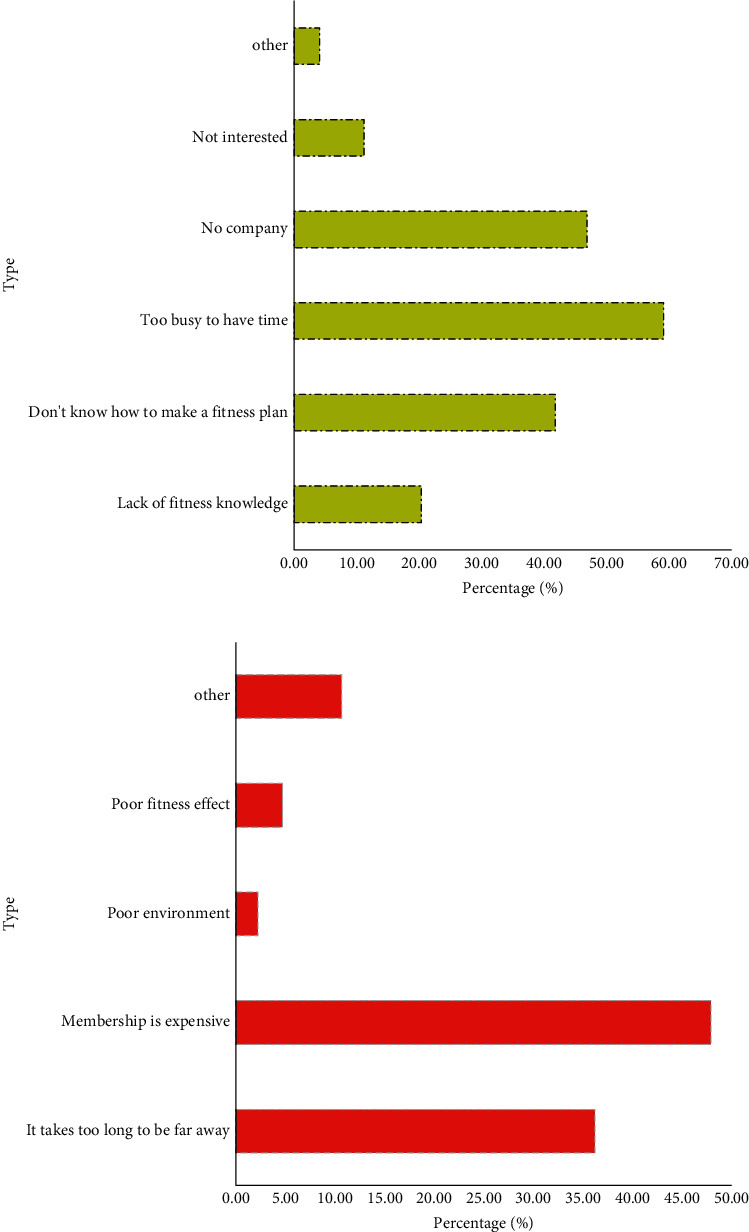
Reasons for not wanting to exercise versus not going to the gym. (a). Reasons for not wanting to exercise. (b). Reasons for not going to the gym.

From the survey statistics of the reasons for not exercising given in Figure 7(a), it can be seen that the reason for about half of the respondents not exercising was that they were busy with work and did not have enough time to exercise. The second and third reasons were that they did not have a fitness partner or did not know how to develop a fitness plan, respectively.

Judging from the reasons why users were reluctant to go to the gym (as shown in Figure 7(b)), we found that most of people did not go because the location of the gym was not so convenient, and they were reluctant to spend time running a distance to go to the gym after a day's work. The second was that gym memberships were expensive.

**Figure 8:**
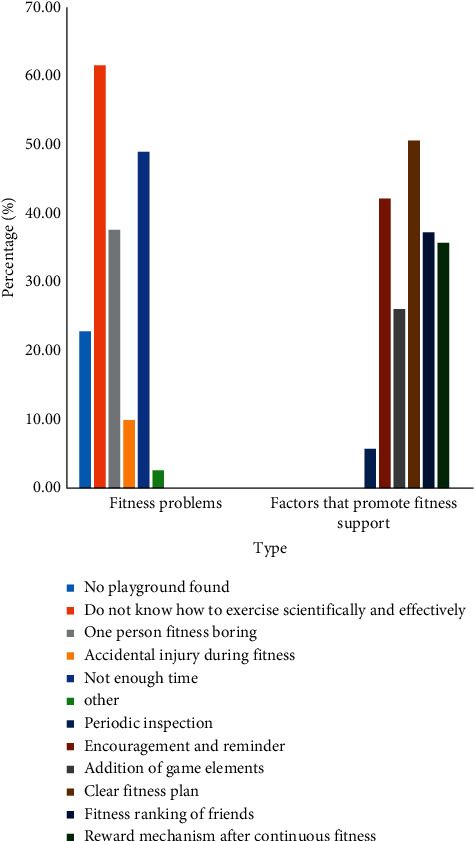
Problems encountered in fitness and factors that promote fitness adherence.

*(3) Fitness Requirements*. In order to understand the needs of target users for fitness, a survey was conducted on the problems encountered by the respondents in fitness. It can be seen from the survey results in Figure 8 that nearly 61.52% of the respondents did not know how to exercise scientifically and effectively. The second reason was that they did not have enough time for fitness. From the survey of factors that promoted fitness persistence in Figure 8, it could be seen that a clear fitness plan and encouragement and reminders in the fitness process could promote the respondents to actively exercise and stick to it.

**Figure 9:**
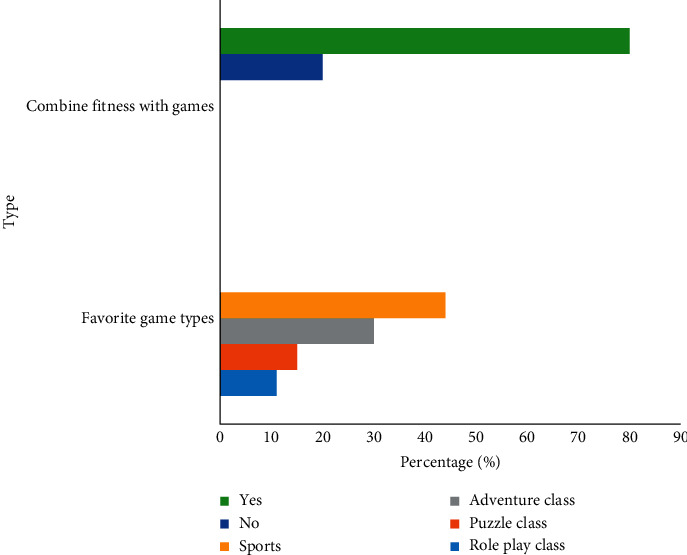
People's favorite game types and the number of people who think it is better to incorporate fitness movements into somatosensory games.

*(4) Research on Somatosensory Games*. From the related questions of the survey on somatosensory games (as shown in Figure 9), it can be known that 31% of the respondents have been exposed to somatosensory games and were willing to continue to try. Finally, further inquiries were made to the respondents who had experienced somatosensory games. 80% of the respondents believed that the integration of somatosensory technology into fitness equipment had a positive effect on improving fitness, such as combining sports with parkour games and so on. Others who believed that somatosensory technology was not helpful for fitness considered that most somatosensory games focused on the exercise of the hands and legs, which was less helpful for common diseases of office workers, such as cervical and lumbar pain, etc. Meanwhile, they also believed that the exercise intensity of somatosensory games was small and could not achieve fitness effects.

### 3.5. Summary of Requirements

1. The office group generally had health problems, especially some subhealth problems. Their understanding of cervical spondylosis and lumbar spondylosis was not very accurate.
2. Users needed accurate fitness action guidance, appropriate fitness equipment design, reasonable fitness time settings, and interesting fitness game content.
3. This group had less time for fitness, and the time is not concentrated, so it was not suitable for going to the gym for systematic training.
4. The main fitness methods of the surveyed users were running, exercising with simple fitness equipment at home, etc.
5. In terms of whether it is acceptable to join the virtual reality technology, most people were acceptable and interested in it, which proved that the research on this topic was feasible and had market prospects.

## 4. Conclusions

With the development of science and technology and the concept of “national fitness,” more and more people begin to pay attention to their own health. People are no longer satisfied with the traditional fitness model, and the addition of high-tech and virtual reality technology makes exercise no longer boring. The progress of society has made most people become office workers, and they do not need to go out to do some physical work, but the long-term desk work that follows has also affected the health of office workers. Therefore, it is necessary and promising to design a virtual fitness product that integrates entertainment and fitness. This paper takes Kinect virtual technology as the technical support and the office family as the target group, summarizing the research status at home and abroad through literature reading, in order to study the characteristics of the target users. Then, with the help of user interviews, questionnaires, field research, and network research, the user's needs and design points are summarized. Finally, through the design practice, the validity of the subject research is illustrated, and the subsequent research on the home fitness products of office family provides a reference. However, due to personal knowledge, time, energy, and other reasons, the research still has shortcomings. As virtual reality technology is more and more understood by people, it is believed that this technology will be applied to more fitness products in the future, enriching the types of fitness products, which will help people to better exercise and maintain their health.

## Figures and Tables

**Table 1:** Functional comparison of virtual reality hardware devices.

	Fixed-end VB	Mobile terminal VB
Equipment	Oculus rift, Samsung Xuanlong, and HTC vive	Camera, software
Output device	Computer, VR helmet	Cloud platform and mobile software
Advantage	Immersive and interactive	Portable and cheap
Shortcoming	Complicated equipment operation and high cost	Immersion and weak interaction

**Table 2:** Arrangement of the physical state of the interviewees.

Interviewee	Sedentary	Physical condition after sedentary	How to adjust
*A*	Yes	Pain of lumbar vertebra and cervical vertebra	Knead by yourself
*B*	Yes	Stiff neck	Laissez faire
*C*	Yes	Discomfort of lumbar vertebra and cervical vertebra	Run
*D*	Yes	Eye fatigue and cervical discomfort	Jump aerobics
*E*	Yes	Cervical discomfort	Manual tapping
*F*	Yes	Shoulder and neck ache	Occasional massage

**Table 3:** Arrangement of the fitness of the interviewees.

Full name	Fitness or not	Current fitness mode	Frequency and time	Place
*A*	Yes	Dumbbell, push-ups, and other simple fitness methods	Two or three times a week, after work	At home
*B*	Yes	Playing basketball, using dumbbell, and other exercises	Once a week, in the evening	Park, home
*C*	Yes	Running, rope skipping, and other fitness methods	Twice a week, at night	Downstairs of the community
*D*	Yes	Run	Twice a week, at night	Park
*E*	Yes	Practice yoga and run by themselves	Once a week, in the evening	Home, park
*F*	Yes	Running, aerobic exercise, etc	Twice a week, at night	Gym

**Table 4:** Arrangement of fitness methods.

Full name	What fitness methods have you tried?	Do you want to go to the gym for exercise? What are the main reasons why you cannot go to the gym?
*A*	Dumbbell and push-ups	Hopes to go to the gym. I often work overtime and have no time to go
*B*	Play basketball and exercise with dumbbells	Hopes to go to the gym. I am busy with my work. It is a long distance away
*C*	Running and rope skipping	Hopes to go to the gym. The price of the gym is high and there is less free time
*D*	Running and using dumbbells	Hopes to go to the gym. I am busy and have no time
*E*	Yoga and running	Hopes to go to the gym. I am busy and have no time
*F*	Run	Hopes to go to the gym. It costs more and has less free time

**Table 5:** User perceptions of joining virtual games.

Full name	Views on adding virtual games to fitness	Whether somatosensory games have effect on fatigue of the lumbar vertebrae and cervical vertebrae
*A*	Like to join the game	Effective
*B*	Like to join the game	Effective
*C*	Like to join the game	Effective
*D*	Dislike	—
*E*	Like to join the game	Effective
*F*	Like to join the game	Uncertain

## Data Availability

The data used to support the findings of this study are available from the corresponding author upon request.

## References

[1] Kidokoro T., Peterson S. J., Reimer H. K., Tomkinson G. R. (2021). Walking speed and balance both improved in older Japanese adults between 1998 and 2018. *Journal of Exercise Science & Fitness*.

[2] Wang Y., Chen S., Zhang J. (2017). Nonexercise estimated cardiorespiratory fitness and mortality due to all causes and cardiovascular disease: the NHANES III study - ScienceDirect. *Mayo Clinic Proceedings: Innovations, Quality & Outcomes*.

[3] Talbot L. A., Thomas M., Bauman A., Manera K. E., Smith B. J. (2021). Impacts of the national your brain matters dementia risk reduction campaign in Australia over 2 years. *Journal of Alzheimer’s Disease*.

[4] Jhang L. Y., Huang H. S., Hsu Y., Liu W. M. (2020). [Lower extremity exercise improves functional fitness, physiological indexes, exercise self-efficacy, sleep quality, and mental health in middle-aged and older individuals]. *Hu li za zhi The journal of nursing*.

[5] Aliyu F., Talib C. A. (2019). Virtual reality technology. *Asia Proceedings of Social Sciences*.

[6] Maples-Keller J. L., Bunnell B. E., Kim S. J., Rothbaum B. O. (2017). The use of virtual reality technology in the treatment of anxiety and other psychiatric disorders. *Harvard Review of Psychiatry*.

[7] Liang Z., Shuang R. (2017). Research on the value identification and protection of traditional village based on virtual reality technology. *Boletin Tecnico/Technical Bulletin*.

[8] Zhang H., Zheng H. (2017). Research on interior design based on virtual reality technology. *Boletin Tecnico/Technical Bulletin*.

[9] Lan L., Fei Y., Shi D., Jiang Q. (2017). Application of virtual reality technology in clinical medicine. *American Journal of Tourism Research*.

[10] Hughes S., Warren-Norton K., Spadafora P., Tsotsos L. (2017). Supporting optimal aging through the innovative use of virtual reality technology. *Multimodal Technologies and Interaction*.

[11] Chen T. N., Yin X. T., Li X. G. (2018). [Application of 3D virtual reality technology with multi-modality fusion in resection of glioma located in central sulcus region]. *Zhonghua Yixue Zazhi*.

[12] Lai P., Zou W. (2018). The application of virtual reality technology in medical education and training. *Global Journal of Information Technology Emerging Technologies*.

[13] Zeming L. (2017). Design and implementation of a Korean language teaching system based on virtual reality technology. *Agro Food Industry Hi-Tech*.

[14] Lee H. S., Lee J. H. (2020). The effect of T-ball class on physical self-efficacy of elementary school students using virtual reality technology(VR). *Korean Journal of Sports Science*.

[15] Meng N.  (2020). Application of intelligent virtual reality technology in Clothing virtual wear and color saturation after COVID-19 epidemic situation. *Journal of Intelligent and Fuzzy Systems*.

[16] Wodzyski M. (2020). Design of a research and training platform for operating portable chainsaws using virtual reality technology. *Problems of Mechatronics Armament Aviation Safety Engineering*.

[17] Feng C. (2020). An intelligent virtual reality technology in the teaching of art creation and design in colleges and universities1. *Journal of Intelligent and Fuzzy Systems*.

[18] Peng Y., Lyu L. Y., Ma B. (2020). [Advances in the research of application of virtual reality technology in war trauma treatment training]. *Zhonghua shao shang za zhi = Zhonghua shaoshang zazhi = Chinese journal of burns*.

[19] Zhang R., Zhao X. (2020). The application of folk art with virtual reality technology in visual communication. *Intelligent Automation & Soft Computing*.

[20] Wells T., Miller G. (2020). The effect of virtual reality technology on welding skill performance. *Journal of Agricultural Education*.

[21] Wu W. W., Liu S. M., He T. T., Wu S. F. (2020). [Advances in the research of virtual reality technology for pain intervention after burns]. *Zhonghua shao shang za zhi = Zhonghua shaoshang zazhi = Chinese journal of burns*.

[22] Zel S., Kongar E. (2020). Influential factors in design and implementation of virtual reality technology[j]. *MATTER: International Journal of Science and Technology*.

